# Transgene copy number estimation and analysis of gene expression levels in *Populus *spp. transgenic lines

**DOI:** 10.1186/1753-6561-5-S7-P152

**Published:** 2011-09-13

**Authors:** Francesca Donnarumma, Donatella Paffetti, Matthias Fladung, Stefano Biricolti, Ernst Dieter, Illimar Altosaar, Cristina Vettori

**Affiliations:** 1 Plant Genetics Institute, CNR, Italy; 2 Department of Agricultural and Forest Economics, Engineering, Sciences and Technologies, University of Florence, Italy; 3 Johann Heinrich von Thuenen Institute (vTI), Forestry and Fisheries Institute for Forest Genetics, Germany; 4 Dipartimento di Ortoflorofrutticoltura, Università degli Studi di Firenze, Italy; 5 Helmholtz Zentrum München, German Research Center for Environmental Health (GmbH), Germany; 6 Department of Biochemistry, Microbiology and Immunology, University of Ottawa, Canada

## Background

The genus *Populus* has certain important features, such as a relatively small nuclear genome, it can be easily regenerated easily *in vitro* and genetically transformed by *Agrobacterium* vector system, which make it ideal for gene transfer and molecular genetic studies in forest trees [1]. Insect-tolerant poplars have been obtained using several types of insecticidal genes coding for *Bacillus thuringiensis*-toxins. Regenerated plants with insect-resistance were obtained in different studies. *Agrobacterium*-mediated transformation has been the favored method for the introduction of foreign genes into plants. The effectiveness of insect-resistance in transgenic plants is related to the side effects of gene transfer (site of gene insertion, copy number, gene silencing etc.).Moreover intransgenic plants, transgene copy number can greatly affect the expression level and genetic stability of the target gene, making estimation of transgene copy numbers an important area of genetically modified plant research [2]. Thus molecular biological analysis of transgenic plants, like real time PCR, widely used to detect and quantify DNA and cDNA [3], could represent an useful tool to investigate the genetic stability of transgenic forest trees having a long life cycleas well as for determining copy number in transformed plants.

## Material and methods

The present study was undertaken to investigate *Populus alba* and *P. tremula* x *P. tremuloides* transgenic lines, obtained via *Agrobacterium*-mediated transformation, carrying *cry1Ab* and *nptII* genes in the T-DNA region. The plants were vegetatively propagated in growth chambers over 2 years. Ten individuals from each clone were planted in containers with "forest soil", and grown in a climate chamber.

Extraction of genomic DNA and RNA from leaves was performed for PCR and Real Time PCR (RT-PCR) analysis to estimate the transgene copy number [4] as well as expression of the inserted gene [5]in transgenic poplar, respectively.

## Results and discussion

**Figure 1:**
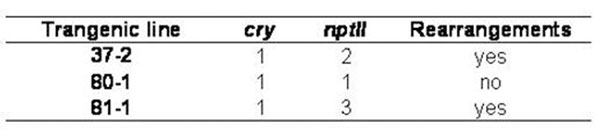
Copy number values estimated by real-time PCR. Data are expressed as mean ± 95% confidence limit.

All lines contained one copy of *cry* gene and two of them showed that the copy number was different for the *cry1Ab* and *nptII* genes, suggesting rearrangements or multiple but incomplete copies of the transferred DNA (Figure 1). The copy number was concordant among the 3 individuals of each lines analysed and with those determined from the same transgenic lines kept in micropropagation for 2 years.

**Figure 2:**
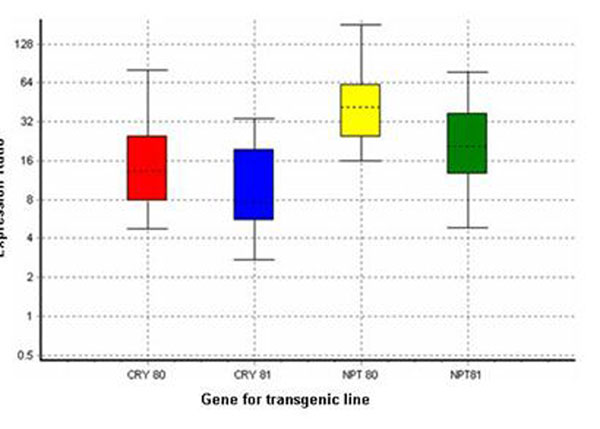
Relative expression obtained through qRT-PCR for the transgenes *cry1Ab* and *nptII* in transgenic *Populus* lines. The data are shown relative to the endogenous *actin* gene. Boxes: interquartile range, or the middle 50% of observations; dotted line: median gene expression; whiskers: minimum and maximum observations. Data are expressed as mean ± 95% confidence limit after 10000 permutations and have a *p* = 0.000 – 0.002.

The transcript levels from both genes were determined in 3 individuals for each line growing in climatic chambers. High levels of mRNA expression were detected with respect to the stable endogenous *actin* gene for both transgenic lines (Figure 2). Comparing the transcript level of inserted genes among lines, a significant low level of *nptII* gene (*p* = 0.005) in the line carrying 3 copies was observed.

Preliminary results indicate a differential expression of endogenous genes among transgenic lines and towards their isogenic form.

## Conclusions

The evaluation of the copy number of the inserted genes has indicated their stability after 2 years of micropropagation. The lower expression level of the *nptII* inserted gene in one line could suggest that factors like position effects or DNA rearrangements lead to differential expression.

The screening of the transcriptomic variations in transgenic plants carrying the *cry* gene and the comparison with position effects or DNA rearrangements is in course. The final aim is to unravel possible pleiotropic transcriptomic effects following *cry* gene expression in *P. alba* and *P. tremula x P. tremuloides* transgenic lines.
